# Understanding barriers to treatment-seeking in Mseleni joint disease: A multistaged study in rural KwaZulu-Natal, South Africa

**DOI:** 10.4102/phcfm.v18i1.5297

**Published:** 2026-05-13

**Authors:** Elizabeth S. Dinkele, Robea Ballo, Victor Fredlund, Victoria E. Gibbon

**Affiliations:** 1 Department of Human Biology, Faculty of Health Sciences, University of Cape Town, Cape Town, South Africa; 2 Mseleni Hospital, KwaZulu-Natal, South Africa

**Keywords:** South Africa, Mseleni joint disease, rural, endemic, treatment-seeking, arthritis, chronic pain

## Abstract

**Background:**

Mseleni joint disease (MJD) is a degenerative chondrodysplasia of unknown aetiology that is endemic to uMkhanyekude in KwaZulu-Natal, South Africa. Delayed treatment-seeking for MJD results in severe joint pain, which commonly progresses to permanent loss of mobility and reliance on caregivers.

**Aim:**

This study aimed to identify systemic, social and cultural barriers to MJD treatment-seeking from the perspectives of patients and healthcare providers.

**Setting:**

The research was conducted at the Mseleni Hospital in KwaZulu-Natal, South Africa.

**Methods:**

A multistage mixed methods study was conducted. Stage One involved quantitative analysis of patient medical records (*n* = 53) and administration of questionnaires to patients (*n* = 37). Stage Two involved a thematic analysis of interviews with MJD patients (*n* = 6), nurses (*n* = 7) and treating MJD (*n* = 9). Findings from both stages were analysed thematically to barriers to treatment-seeking for MJD.

**Results:**

Treatment-seeking barriers fell into three domains: (1) current approaches to treatment and self-management, (2) aetiological perspectives and (3) systemic barriers to healthcare access. Treatment and self-management through traditional medicine and delays in the primary healthcare system were cited as reasons for latent treatment-seeking. Healthcare providers highlighted structural barriers including unstandardised record-keeping, poor cross-facility communication and limited resources, while MJD patients emphasised failed treatments, pain and immobility and inadequate care as key obstacles. Variable aetiological perspectives between MJD patients and healthcare providers were reflected in appraisals of treatment. Gender disparities in sociocultural expectations and stigma-linked to symptoms restricted women’s access to care.

**Conclusion:**

Differing perspectives between MJD patients and healthcare providers contributed to delayed treatment-seeking and hindered the management of MJD. Patients discussed barriers to MJD relative to traditional knowledge, beliefs and lived experience, while healthcare providers discussed barriers relative to systemic and structural factors through a biomedical lens.

**Contribution:**

This study highlights the need to align clinical approaches to treating MJD with patient and provider experiences of this disease to address both individual and systemic barriers to treatment.

## Introduction

**FIGURE 1:**
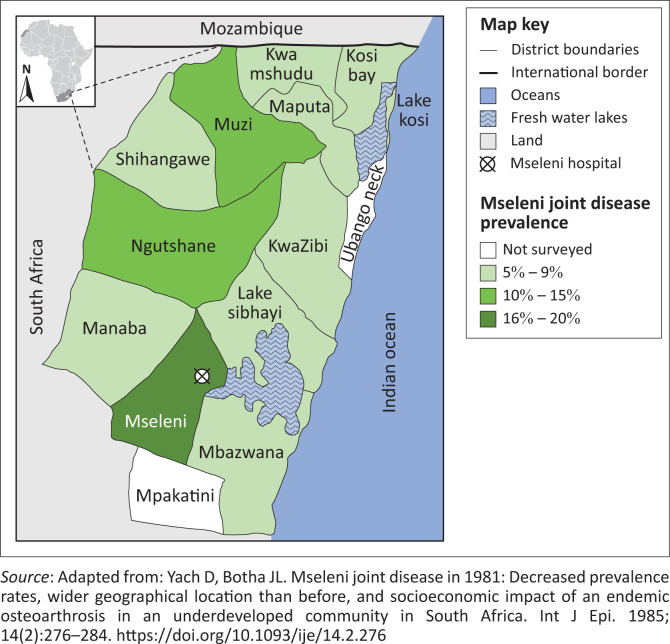
Geographic distribution of Mseleni joint disease assessed per household.

Mseleni joint disease (MJD) is a bilateral, degenerative condition that is primarily localised to the hip joints and causes chronic pain and physical disability in those affected.^1^It is endemic to the rural region of uMkhanyakude in KwaZulu-Natal, South Africa, where it is known to locals as *unyonga* – meaning ‘a disease of the joints’.^2^ It was clinically named MJD because of the localisation of sufferers around and near the Mseleni Hospital (Figure 1^3^).^4,5,6^ The onset of MJD occurs commonly during childhood or adolescence, with first symptoms including joint pain, stiffness and crepitus (clicking sounds during movement).^7,8^ While several genetic,^9,10^ cultural^4,11^ or environmental^4,12,13^ causes have been explored thus far, the aetiology remains unknown. This has led to the suggestion of a multifactorial aetiology, comprising environmental, biocultural and epigenetic factors.^1^ More than 83% of households in the MJD-affected region of uMkhanyakude live below the poverty line, and over 70% of the population subsists on less than R800.00 ($43.00) per month.^14^ Approximately 73% MJD-affected households are fully reliant on subsistence from government pensions and social assistance grants, with care provision falling to young women in families.^15^ This phenomenon results in limited education, which further perpetuates unemployment, poverty and gender inequality in MJD-affected families.^11^

Extensive delays (often up to 17 years^15^) have been reported between the symptom onset and diagnosis – resulting in severe pain, immobility and loss of independence among young adults affected by MJD.^6^ MJD management follows a stepped-care model, in which the safest and most cost-effective treatments are prioritised, with invasive and costly treatments reserved for patients who fail to benefit from initial care.^2^ First-line treatment includes pain management with non-steroidal anti-inflammatory drugs (NSAIDS), followed by physiotherapy to manage pain and preserve joint function.^16^ Total joint replacement is only considered as a final option to improve pain and mobility. Although total joint replacements have been effective in restoring mobility in MJD patients,^4^ its high cost limits widespread use. Estimates suggest that over 2000 patients are currently on the surgical waiting list at the Mseleni Hospital. It would take more than 60 years to treat all patients.^15^ This highlights that total joint replacement is not a sustainable disease management strategy in this setting.^15^ Consequently, current recommendations, therefore, emphasise early detection, physiotherapy and rehabilitation.^2^ This approach reduces disease burden, by slowing functional decline, decreasing reliance on total joint replacement and improving surgical outcomes because of improved pre-operative physical status. Despite the availability of early interventions, these are not often utilised because of delays in seeking treatment.^14,15^ Treatment-seeking behaviours are complex, multifaceted and deeply embedded in regionally unique contextual factors and social dynamics. How individuals perceive, understand and respond to illness reflects prior illness experiences, knowledge systems, belief systems, and broader economic and sociocultural contexts.^17^ These models are not limited to patients; they are shared by all stakeholders engaged in healthcare (including clinicians, nurses and caregivers).^18^ For individuals living with a chronic disease, appraisals of symptoms, assumptions about causation, and responses to medical advice are conditioned by personal experiences and observation of others with similar conditions.^19^ Treatment-seeking and healthcare engagement also reflect perceptions of the healthcare system and accessibility of care. Conventional approaches to studying treatment-seeking often prioritise biomedical constraints such as treatment efficacy, geographic access, or adherence and under-examine social and cultural contexts.^18^ When interventions address biomedical barriers without engaging these contextual factors, they frequently fail to achieve sustained and equitable coverage.^18,20^ Current evidence suggests the rising incidence from chronic diseases in rural regions in South Africa has placed increasing pressure on healthcare services.^21^ Non-communicable diseases (NCDs) are projected to increase globally by 2050, with joint diseases estimated to be the leading cause of disability with a 200% increase in case numbers projected for sub-Saharan Africa.^22,23^ As the chronic diseases burden increases, providing affordable and effective care to the large and increasing numbers of people will be an immense challenge. Considering the paucity of research exploring the array of contextual factors that shape treatment-seeking for MJD, the aim of this study was to explore the barriers to treatment-seeking from the perspectives of patients and healthcare providers. We designed a mixed-methods study with a triangulation of qualitative and quantitative data from medical records, surveys and interviews to identify systemic, social and cultural barriers to care provision in the context of MJD.

## Research methods and design

### Study design

**FIGURE 2:**
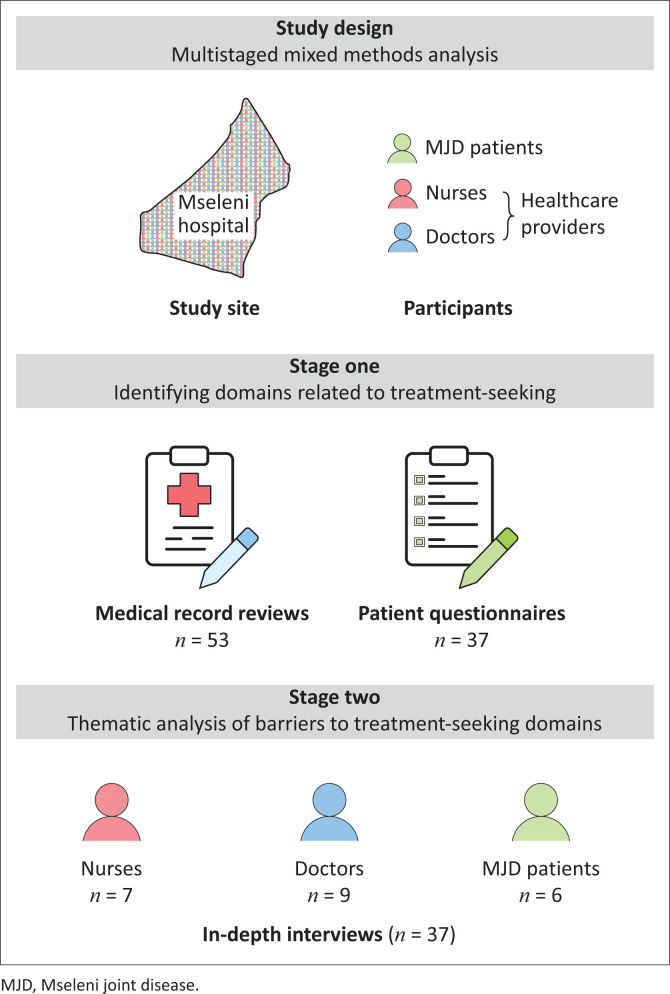
Schematic diagram of the multistaged mixed-methods analysis of treatment-seeking associated with Mseleni joint disease.

We conducted a multistaged mixed methods study incorporating qualitative and quantitative data from medical record reviews, patient questionnaires, and interviews. Figure 2 provides an illustration of the multiple stages in this study. Stage One included medical record reviews and patient questionnaires of MJD patients at the Mseleni Hospital. These data were analysed to identify patterns in onset, diagnoses and treatment-seeking information from healthcare providers and patients’ perspectives, respectively. Stage Two involved in-depth interviews with MJD patients, nurses and doctors to triangulate barriers to treatment-seeking identified in Stage One.

This study was undertaken at the Mseleni Hospital, which is a state-funded district hospital that services more than 90 000 people living in an area spanning 30 000 km^2^. Individuals with joint pain and immobility (symptomatic of MJD) commonly report to local clinics where they are treated by nurses and/or referred to doctors at the clinic or Outpatients Department at the hospital.^2^ The Outpatients Department manages a weekly MJD clinic, hospital admissions, emergency care, day-clinic visits, patient transfers and appointments with therapists, radiologists, doctors and surgeons. Patients and healthcare providers sampled in our study were recruited from the Outpatient’s Department of the Mseleni Hospital.

### Data collection

#### Stage One

Medical records from the Outpatients Department at the Mseleni Hospital in May 2019 were screened in July 2019 to identify MJD patients. Due to the absence of International Statistical Classification of Diseases and Related Health Problems (ICD-10 codes) in the medical records at the Mseleni Hospital, cases of MJD were identified using diagnoses from clinicians under the terms: ‘Bilateral joint disease’, ‘MJD’ or ‘MJD diagnosis’. From these records, descriptive clinical information were extracted, including documented disease duration, recorded signs and symptoms, and treatments prescribed or received during routine care. These data were used solely to characterise patterns of healthcare use and clinical management, not for inferential analysis. Questionnaires were developed based on instruments used in previous MJD studies and included a combination of closed and open-ended questions.^6,7,12^ The questionnaire is provided in supplementary material. Using convenience sampling, MJD patients seeking care at Mseleni Hospital in July 2019 were recruited. To ensure a diverse MJD sample, this group was not restricted to patients identified in the May 2019 Outpatients Department medical records review records, nor was there any overlap. Informed consent was obtained in isiZulu prior to administration of questionnaires with the help of a translator. All semi-structured surveys occurred in isiZulu with the help of a translator. A total of 37 patients consented to and completed the questionnaire. Post-administration debriefs were conducted with the researcher and translator for the first 10 questionnaires and one of the investigators to ensure consistency between patient responses, translations and transcriptions. Findings from the questionnaires were used to identify preliminary themes related to treatment-seeking, which informed the qualitative sampling and interview guides in Stage Two.

#### Stage Two

Based on the domains identified from Stage One, in-depth interviews were conducted with MJD patients (*n* = 6), doctors (*n* = 9) and nurses (*n* = 7). Participants were purposively sampled to capture perspectives of both treatment-seekers and treatment providers at Mseleni Hospital. Written informed consent was obtained from all participants prior to interviews. Doctors and nurses were interviewed in English by a researcher, while patients were interviewed in isiZulu by the same researcher, with the assistance of a trained translator who was locally known to the community. Interviews took place at participants’ locations of choice: patients were interviewed in their homes, and doctors and nurses at Mseleni Hospital. All interviews were audio-recorded using an Olympus^®^ (VN-541PC) digital voice recorder. Recordings were translated into English (for patients) by two independent translators and then transcribed. Interviews lasted between 30 min and 90 min. To ensure accuracy, transcripts were cross-checked by two individuals, and discrepancies were resolved through discussion with co-authors until consensus was reached.

### Data analyses

Quantitative data from medical records and surveys were assessed statistically using χ^2^ tests of association between disease durations, age of diagnosis and patterns of treatment-seeking for MJD. Qualitative data from questionnaires and interviews were analysed within a thematic framework as proposed by Clarke et al.^24^ Using this method permitted the organic identification of themes in qualitative data that reflected patterns in responses or meaning – allowing the underlying research question to be addressed more thoroughly.

*A priori* codes were developed collaboratively by the primary researcher and principal investigator – informed by findings from the medical record review and questionnaires in Stage One. Open coding was then applied to the interview transcripts to identify emergent concepts and themes from the interviews. The steps in coding, sorting and merging, and identifying themes were applied systematically to maximise the validity and reliability of each subsequent step.^24,25^ Following the initial coding process, first-order concepts were grouped into second-order themes based on participants’ emphasis of concepts; these were then grouped accordingly. Methodological and investigator triangulation were used to promote validity of coding and theme development. Methodological triangulation was accomplished through a comparison between different data sources and their representation of participant groups, while investigator triangulation involved coding, thematic development and discussion between multiple members of the research team.^24^

### Ethical considerations

Ethical clearance to conduct this study was obtained from the University of Cape Town Human Research Ethics Committee (HREC: 079/2019), Mseleni Hospital, the District Health Authority and the KwaZulu-Natal Provincial Department of Health (KZ_201902_002). Ethical guidelines in the Declaration of Helsinki (2008) and the South African Department of Health: Ethics in Health Research: Principles, Structures and Processes (2004) were followed in this study.

## Results

MJD was diagnosed in 66 out of 1409 patients. Of these MJD patients approximately 37 came to the MJD clinic in May 2019. Complete medical records could only be obtained for 53 MJD patients. The sample comprised more medical records from women (70%, *n* = 37/53) than men (30%; *n* = 16/53). The MJD patients sampled in medical records were largely skewed towards old age. Specifically, 26% (*n* = 22/53) were older than 71 years-of-age, and 19% (*n* = 14/53) were 61–70 years-of-age. In medical records, approximately, 42% (*n* = 22/53) of MJD patients had disease durations of 0–5 years (Table 1), with 13% (*n* = 7/53) and 9% (*n* = 5/53) with disease durations of more than 6–15 years. The survey sample of MJD patients was also skewed in representation of women (92%, *n* = 34/37), and individuals over 61 years-of-age (Table 1). Approximately, 56% (*n* = 20/37) of surveyed patients described onset compared to an age or year while 28% (*n* = 10/37) described onset compared to stage-of-life, and 17% (*n* = 6/37) according to a significant life event (Table 2). In-depth interviews were conducted with 6 MJD patients, 7 nurses and 9 doctors. More interviewed patients were female (*n* = 4) than male gender (*n* = 2). Patients had a mean age of 69 years-of-age and a range between 58- and 76-years-of-age. Doctors were mostly of male gender (*n* = 6/9) and had been practicing for 2–25 years at the Mseleni Hospital. Nurses were primarily of female gender (*n* = 6/7) with an average of 10–30 years of experience at the Mseleni Hospital.

**TABLE 1:** Participants’ characteristics from medical records and the semi-structured surveys assessed quantitatively.

Variables	Medical records (Patients [*n* = 53])		Questionnaires (Patients [*n* = 37])		Interviews		
	*n*	%	*n*	%	Patient (*n* = 6)	Nurses *(n* = 6)	Doctors (*n* = 9)
**Gender**							
Women	37	70	34	92	4	4	3
Men	16	30	3	8	2	2	6
**Age (years)†**							
31–50	7	13	5	13	0	-	-
51–60	21	18	10	27	1	-	-
61–70	14	19	11	30	2	-	-
71+	22	26	11	30	3	-	-
**Disease duration (years)** ‡							
0–5	22	42	12	32	0	-	-
6–15	7	13	6	16	4	-	-
16+	5	9	17	46	2	-	-
Not recorded	19	36	2	5	0	-	-

Note: Percentages calculated within columns.

*n*, number of individuals; %, percentage.

† , 11 patients did not answer;

‡ , Disease duration calculated from the onset of symptoms to the time of the survey/medical record review/interview.

**TABLE 2:** Frequency distributions of clinical assessments, symptoms and treatment of Mseleni joint disease recorded from medical records and questionnaires.

Variables	Medical records (*n* = 53)		Questionnaires (*n* = 37)	
	*n*	%	*n*	%
**Signs and symptoms†**				
Joint pain	53	100	-	-
Swelling present	8	15	-	-
Radiating leg pain	31	59	-	-
**Treatment**				
Physiotherapy				
Adherence	41	78	11	30
Non-attendance	1	2	17	46
No referral	11	21	9	24
Pain management				
NSAIDS	52	98	29	78
None	5	14	3	8
Surgical joint replacements				
Total joint arthroplasty (TJA)‡	12	22	6	15
Multiple TJAs	4	8	2	6
Non-attendance	4	8	12	32
Operation scheduled	4	8	-	-
No referral	29	55	7	19
**Causes**				
Environmental				
Soil	-	-	7	19
Farming and food source	-	-	1	2
Environmental pollutants	-	-	1	2
Lifestyle				
Labour and activities	-	-	5	14
Spiritual				
Witchcraft	-	-	4	11
Accidental injuries	-	-	1	2
Unsure	-	-	18	49

Note: Percentages calculated within columns.

*n*, number of individuals; %, percentage; TJA, total joint arthroplasty; NSAIDS, non-steroidal anti-inflammatory drugs.

† , Symptoms as recorded or reported during survey period;

‡ , No answer provided by 11 patients in surveys.

**FIGURE 3:**
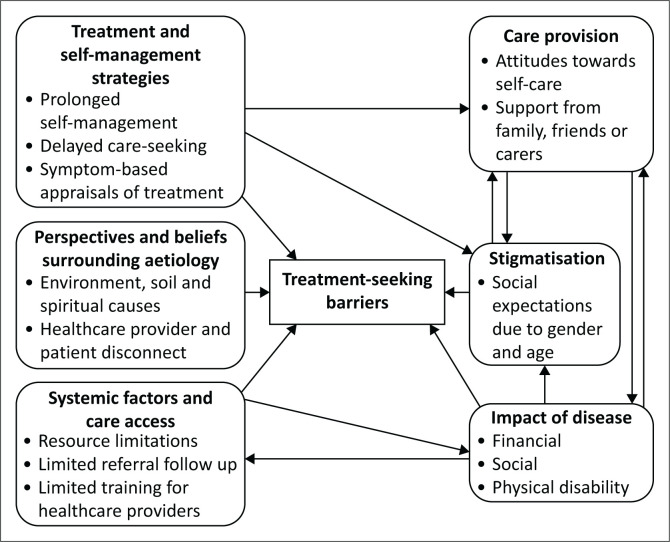
Conceptual model illustrating the link between treatment-seeking domains, care provision, stigmatication and the impact of Mseleni joint disease.

We identified 3 primary domains and several overlapping subthemes linked to treatment-seeking behaviours in patients with MJD (Table 3). These were: (1) current treatment and self-management strategies; (2) perspectives and beliefs surrounding aetiology; and (3) systemic barriers and access to care. In Figure 3, we present a conceptual model illustrating the linkage between these domains and their relationship between individual experiences of MJD inclusive of systematic challenges to seeking treatment.

**TABLE 3:** A summary of domains, themes and concepts associated individual experiences of Mseleni joint disease and systemic barriers to treatment-seeking.

Domain	Theme	Concepts
Current approaches to treatment and self-management strategies	Treatment appraisals	• Appraisals of treatments-linked primarily to pain relief • Analgesia and surgery are preferred to physiotherapy • Rural environment limits treatment efficacy and longevity
	Gender and care provision	• Enabling treatment Gendered differences in care provision • Age-related stigmatisation • *uMakhoti*’s are central to care provision
Perspectives and beliefs surrounding aetiology	Intersecting knowledge systems	• Environmental causes • Biomedicine as a knowledge system • Traditional knowledge systems
Systemic factors and care access	Systemic barriers to treatment seeking exacerbate individual-level barriers.	• Resource limitations of patients (transport, social and financial) • Constraints of the healthcare system (limited resources, poor cross-facility communication, limited training for healthcare providers)

### Current approaches to treatment and self-management strategies

Approximately, 40% (*n* = 21/53) of MJD patients visiting the OPD during the study period reported new sources of pain, and 21% (*n* = 11/53) were given prescription renewals for analgesia (Table 2). From medical records (*n* = 52/53; 98%) and surveys (*n* = 29/37; 78%), it was clear that NSAIDS were the most used treatment for MJD (Table 2). No referrals to physiotherapy were recorded in medical records for 21% (*n* = 11/53) of individuals, and furthermore, non-adherence to physiotherapy was only identified in one patient’s medical record. Self-reporting of non-adherence to physiotherapy was common among surveyed MJD patients (46%, *n* = 17/37), although no significant associations were detected between age and adherence to physiotherapy treatment (Table 2). Approximately 23% (*n* = 12/53) of MJD patients from the medical record reviews had undergone a single joint replacement, while 8% (*n* = 4/53) had multiple operations including revision surgeries (Table 2). Older patients sampled in the medical record review were more likely to refuse surgery than younger patients.

In interviews, patients described seeking treatment for MJD only when their symptoms were resistant to self-management strategies or were disrupting their daily activities. Collectively, these accounts reflect a reactive pattern of care-seeking, in which engagement with formal healthcare occurs only after prolonged self-management and functional decline. Treatment appraisals discussed were symptom-based, with pain relief driving decisions to initiate or discontinue care. As a result, analgesia and surgery were discussed more positively than physiotherapy (Table 3):

‘I don’t think there is anything else that could help me except for the pills I’m already using.’ (Patient, F)

‘I only knew pain that was different, and which was slightly better after surgery …’ (Patient, F)

‘I stopped going [*to physiotherapy*] because it felt like they were just finishing me off when they were doing it.’ (Patient, M)

Both doctors and MJD patients found that surgery resulted in good reductions in joint pain and improved mobility. Patients and doctors believed that the demands of rural environments and lifestyle were limiting to the longevity of prosthetics. Pain-related functional decline was described as loss of independence and livelihood, particularly through reduced ability to garden and farm:

‘You have, prosthesis sizing problems and then you have a, you have a higher demand of a rural life on the joint afterwards.’ (Doctor, M)

‘These metals they put on my hip get sweaty because I spend too much time in the sun. They told me to try and decrease the amount of work I do but I don’t like sitting and doing nothing, my heart longed to do more work.’ (Patient, F)

‘It has affected me greatly because I used to farm a lot. When I was affected, I used to think how am I going to live now? Because farming sustained my livelihood.’ (Patient, F)

‘I can go to garden. But I go with pain, but I still persist, stubbornly so that I can farm or garden maize, samp, nuts … but now I cannot do that because of the pain, as soon as I stand, I have to sit.’ (Patient, M)

#### Gender and care provision

Gendered differences in care networks were evident. Women who completed questionnaires more often attended care alone, while men were accompanied by wives or daughters-in-law at the Mseleni Hospital. One doctor found that younger women with MJD experienced stigma when pain and immobility prevented them from fulfilling household roles. Age and community-wide expectations of women as hardworking and caregivers were cited as a cause of stigmatisation and social disharmony for younger MJD patients:

‘So, if it is a younger population, you may find that they’re not, uh, sympathetic towards those women that are having the condition. Whereas you kind of look to older people with the joint disease and you’re kind of saying, well the gogos’ [*grandmothers or older ladies*] are sitting at home and they’re being cared for …’ (Doctor, F)

‘I think the immobility part becomes sort of the point of stigmatization. For instance, I remember someone in the community had two wives, one of the wives didn’t have MJD and then the other had MJD. So, it was almost like this one [*with MJD*] was the lazy one, just because she couldn’t mobilize.’ (Doctor, F)

Across interviews, daughters and daughters-in-law (*uMakoti*) were identified as primary caregivers. *uMakoti*’s fulfil the role of caregiver within households, thereby enabling and facilitating treatment-seeking for MJD. One patient found that her own daughter could not be expected to provide care as she was *uMakoti* in another family. She described struggling to care for herself prior to receiving assistance from her new daughter-in-law:

‘I don’t except this for my own child, she’s someone’s child [*daughter-in-law*]. What I mean to say is he [*my son*] has a child with this child [*my daughter-in-law*]. She helps me, but before she was here I used to struggle for myself.’ (Patient, F)

### Perspectives and beliefs surrounding aetiology

In open-ended questionnaire responses, patients most commonly cited soil and environmental conditions as a suspected cause of MJD. Interviewed doctors and nurses cited soil as a cause; they hypothesised this was because of mineral deficiencies in locally farmed food or soil contaminants:

‘I don’t know. They, they research, but they say it’s this soil, it’s not having the iron.’ (Nurse, F)

‘What about the food that is growing in the soil? Is the soil setting a problem with the food that we are eating? Because maybe there are toxic things that are already in the food.’ (Doctor, M)

‘They talk about environmental and you are kind of thinking that it’s quite an unusual, um, place to live in because it’s all sand-based. And I’m sure if you’re living here and this is the normal terrain that you’re coping with, it’s probably related.’ (Doctor, F)

While patients also cited soil as a cause of MJD, this was conceptualised and explained in reference to the type, quantity of soil and local beliefs surrounding witchcraft:

‘Ei! I haven’t heard anything apart from the fact that it’s the soil where we live.’ (Patient, F)

‘They [*the community*] say it’s caused by the sand but now there is sand everywhere … So that’s why I don’t believe in that theory. There’s a lot of sand in Manaba compared to here but they are still saying it’s the sand. I don’t believe in that’. (Patient, M)

#### Intersecting knowledge systems

Patients were reluctant to describe traditionally held beliefs surrounding the cause and treatment of MJD, and only one patient cited the belief that MJD was caused by someone in the community cursing them, using *umeqo*. Doctors and nurses were informed about traditional healing beliefs and practices, and provided an explanation of *umeqo* and the use of *caba* as a treatment for MJD (Table 3):

‘Well, this started like this. When it enters someone, they would say that they have Umeqo. You remember right? Yes, something painful will entered my leg. So, we started with the traditional route, do you see these cuts, they did this saying that they were healing us saying that it will end but it never stopped.’ (Patient, F)

‘So umeqo is when a witch basically, casts a spell on a path that you’re gonna’ walk on and as soon as you step over that spell then you may have issues with your legs and mobilization. And. So, it’s likely that as the MJD develops, that can be viewed as some form of witchcraft of sorts.’ (Doctor, F)

‘Okay, yeah, sometimes they think they are being punished by the ancestors and so they will do maybe, ceremonies and other rituals for the ancestors.’ (Nurse, M)

‘Witches, this kind of things is happening. Is that person who did this to me, that person did this to me. So, they want to resolve the problem first in the community.’ (Nurse, F)

### Systemic factors and care access

Review of medical records indicated that all identified MJD patients had been diagnosed using radiographs; however, no written radiographic descriptions or interpretive reports were documented in the clinical files. In addition, there was no evidence of a standardised diagnostic protocol for assessing MJD across patient records. Considerable inconsistencies in the documentation of pathology, diagnostic assessments, and disease progression were observed, which limited the depth of quantitative analyses that could be conducted using these data. During interviews, some doctors also reported limited formal training in the diagnosis and management of MJD, expressing uncertainty in distinguishing MJD from more generalised arthropathies based on available clinical information. These findings highlight variability in diagnostic practices and documentation for MJD within the hospital setting:

‘I’m not sure exactly how to actually diagnose MJD compared to other joint diseases. So I think we’ll do like a basic arthritis work up, usually X-rays, rheumatoid factor, inflammatory markers and then, depending on what you find on that, if there’s an obvious breakdown in the joint, then we will discuss with Doctor X or we’ll discuss it with Orthopedics at Ngwelezana.’ (Doctor, M)

‘There wasn’t a specific orientation regarding it, but as it’s in the roster, and there are operations done, and you’ve seen patients in OPD, you kind of find out about it. So, it was more learn as you go type of thing.’ (Doctor, M)

During interviews, doctors described how late presentation as a critical issue in treating MJD and cited systemic causes (fragmented record-keeping, geographic and transport barriers) that interact with these individual-level challenges such as pain, immobility and a lack of support (Table 3):

‘If they’re having problems, severe problems walking, they may end up being referred.’ (Doctor, F)

‘They’ll pick them up, send them for an X Ray. Then that’s when we pick them up, they come you, you go into the old notes you find out that they’ve been complaining about joint pain. And then they also present late, you know. Late presentation, when you do X-ray, you’ll see that the joints just destroyed and then there’s nothing that you can do that to try to give them hope of walking again.’ (Doctor, M)

‘The mobile clinic is going there, and then if there are nurses who can give the patient medication. You see how you are hiding the condition. So, the time you’ll be worse, is the time when you’ll come to the hospital.’ (Doctor, M)

‘So it depends where the patient lives, distance from the hospital, some live far away. Then obviously they can’t walk, they would rely on someone else taking them to the hospital. And if they don’t have transport, they’re not going to come here.’ (Doctor, F)

## Discussion

Previous work on the management of MJD has primarily focused on understanding the aetiology of this disease^1,4,5,6,8,9,12,16,26,27,28,29,30^ and its social impact.^11,15^ This study is the first to examine treatment-seeking behaviours in MJD integrating patient and healthcare-provider perspectives through medical record reviews, surveys and interviews. We identify three interrelated categories of barriers to treatment-seeking: (1) current treatment and management strategies (including perceptions of pain, limited uptake of physiotherapy, and constrained surgical options); (2) aetiological and belief-based barriers, shaped by divergent biomedical and cultural explanatory models of MJD; and (3) systemic and structural barriers, including transport costs, inconsistent clinical record-keeping, and limited continuity of care. By delineating these barriers, this study provides a framework for targeted, context-specific interventions to improve MJD management.

### Current approaches to treatment and self-management

Individuals sampled in our study used various combinations of treatments available for MJD – analgesia, physiotherapy, joint arthroplasties and traditional medicines. From both clinical records and patient surveys, it was clear that pain management with analgesia was most utilised by MJD patients, while physiotherapy was the least utilised treatment. Patients consistently appraised treatments according to their ability to relieve pain, resulting in favourable views of analgesia and surgery, while physiotherapy was often perceived as harmful as a result of short-term increases in pain. These findings align with evidence that individuals with arthritis commonly avoid physical activity because of fears of worsening symptoms, favouring pharmacological or surgical approaches instead.^31,32^ The reliance on symptom-based appraisals has important implications for disease progression in MJD. While physiotherapy plays a critical role in maintaining mobility, reducing pain and improving surgical outcomes, its perceived association with discomfort appears to reinforce prolonged self-management and delayed engagement with care. These findings highlight a disconnect between clinical recommendations and patient perceptions, underscoring the need for contextually appropriate education around the preventive role of physiotherapy in MJD. The MJD patients undergoing joint hip replacements at the Mseleni Hospital have reported good reductions in pain and recovery of joint mobility reduced pain and mobility.^2,15^ Interviewed doctors, confirmed this, although they believed that the demands of rural lifestyle were limiting to the longevity of prosthetics. Fredlund,^2^ reported that patients between the ages of 30 years and 40 years who received joint replacements experienced marked improvements in mobility; however, their prostheses more quickly experienced failure because of considerable physical demands of rural environments. Few patients had multiple joint replacements (8% from the medical records and 6% from the questionnaires), which may be indicative of factors such as age, fear of surgery and limited post-operative. Interviewed patients were predominantly older (mean age 69 years) in our study and had long disease durations, reflecting perspectives shaped by advanced disease. This indicates a critical gap in understanding treatment-seeking behaviours earlier in the disease course and highlights the need for future studies focused on younger patients with MJD.

### Perspectives and beliefs surrounding aetiology

Aetiological beliefs surrounding MJD differed substantially between patients and healthcare providers. Doctors and nurses commonly cited soil-related causes, hypothesising that these were because of mineral deficiencies in locally farmed food or environmental contaminants. In contrast, patients described soil as a cause within culturally embedded frameworks involving the type and quantity of soil and beliefs related to witchcraft. Within traditional African medicine, being healthy and prosperous requires living in harmony with ones *umhlaba* [world or soil], *umhakathi* [community], *isintu* [culture] and *ingqikithi* [the soul].^33,34^ Interestingly, within this paradigm, MJD is believed to be caused by disharmony with one’s soil and community. The MJD was described as a condition caused by *umeqo*, which is caused by walking over soil containing the harmful creation of a sorcerer.^34^ Symptoms of *umeqo* include joint pain and limited range of movement – chronic symptoms of MJD. Treatment for *umeqo* is called *caba*, which involves administering traditional medicines and herbs through incisions made at painful joints in an attempt to remove and treat ‘bad blood’.^34^ While patients, doctors, and nurses were aware of traditional practices such as *caba*, only one patient openly discussed personal use. Patients were often reluctant to articulate spiritual explanations during interviews, yet freely discussed biomedical care received at the hospital. This suggests that patients may selectively disclose beliefs depending on perceived power dynamics and institutional affiliations. Traditional explanations of illness in South Africa are deeply linked to land, ancestry, and community, and may not be readily shared with those who do not share this context.^34,35,36^ This phenomenon is not unique to MJD and has been reported in people of isiZulu-language in different regions in South Africa,^34,35,36^ emphasising the need to further understand the sociocultural patient-provider dynamics involved in the treatment of MJD.

### Systemic factors and care access

Healthcare providers identified inconsistent record-keeping, limited specialised equipment and high patient volumes as major systemic barriers to MJD management. These systemic constraints are linked to individual-level barriers, contributing to delayed presentation and fragmented follow-up. From medical record, a lack of consistency in record keeping was evident between clinicians and across visits. One recommendation towards improving this would be the introduction of a proforma detailing diagnosis, progression and management of MJD. This simple and cost-effective solution may indeed improve patient management and promote treatment-seeking for MJD. This has already been successfully achieved in South Africa, as demonstrated by HIV and TB management programmes, as more consistent care and communication has been linked to adherence to disease management plans.^33,37^ Despite the availability of free healthcare at the Mseleni Hospital, access remains constrained by transport and physical immobility. Radiographic services are only available at the hospital, requiring travel that is often unaffordable or physically taxing for patients with advanced joint disease. Transport accessibility is a hidden barrier to care - particularly impactful in rural regions in South Africa. Limited staffing and poor cross-facility communication further delay referrals and follow-up. While these challenges are not unique to MJD, they substantially impede effective intervention in this setting. Care provision for MJD extends beyond the clinical setting and is deeply embedded within family and,^6,15^ this study is the first to explicitly describe the role of *uMakoti* [daughters-in-law] in facilitating treatment-seeking for MJD. *uMakoti* is an isiZulu word that directly translates as ‘the bride’ or ‘the wife’, and commonly refers to a daughter-in-law (the prefix ‘u’ denotes a definitive article). *Makoti* is not simply a title; it is a position of significant status within the community and household.^33^ The designation of an *uMakoti* carries a set of cultural expectations, which include being a ‘good’ woman, wife, mother, and in-law – demonstrated through caring, hard work at home, obedience to male authority figures and being well respected in the public domain.^33^ Age emerged as a critical determinant of these expectations, with younger *uMakoti* bearing a disproportionate caregiving burden. Healthcare providers interviewed in our studies described stigma and social disharmony affecting younger women with MJD who were unable to fulfil these roles because of pain and immobility. Men with MJD interviewed in our study were accompanied in interviews by *uMakoti’s*, suggesting that they likely had more extensive support and care networks than the interviewed women – although it must be observed that this sample of men was relatively small (*n* = 2). Exploring the treatment-seeking behaviours, and disease outcomes of *uMakoti’s* with MJD should be prioritised towards implementing interventions that provide for care-needs patients with this condition, regardless of their gender. Such stigma may further delay care-seeking and worsen disease outcomes among younger women. While *uMakhoti’s* are central to care provision, they are not typically healthcare-decision makers. One study caregivers to TB-patients reported that *uMakhotis* were more likely to defer treatment decisions to male authority figures, including doctors.^33^ This dynamic would be worth exploring in the case of MJD, given that several doctors at the Mseleni Hospital are men. Understanding gendered power dynamics is essential for designing interventions that support both patients and caregivers within households.

### Study’s limitations

Our findings may not be directly translatable to other osteoarthropathies or sociocultural contexts. The study design involved three data sources – medical record reviews, questionnaires and in-depth interviews – and it was not possible to link these data sources at the individual patient level. As a result, we cannot assume consistency of experiences, beliefs or treatment histories across all participants represented in the different datasets, and findings should be interpreted as complementary rather than individually longitudinal. In addition, we acknowledge that our sample represents a cohort of patients with MJD who sought care at the Mseleni Hospital and is thus biased towards individuals employing more active coping strategies. Interpretation of disease duration of participants of this study should be approached with caution, as medical records reflect duration of documented care rather than symptom onset, while interview may reflect memories of symptoms that occurred several years prior. As such, these may not necessarily be comparable. Information on comorbidities (e.g. human immunodeficiency virus [HIV], tuberculosis [TB], obesity or smoking status) was not collected as this was not the focus of the study. However, given the high burden of chronic comorbidities in this setting, future research should explicitly examine how coexisting conditions influence MJD progression, treatment-seeking behaviour, and outcomes, as this remains an important gap in the literature. As researchers were not locals to the region, and it is possible that study participants were cautious about what they said to the researchers, leading to reporting bias. While we worked from translated verbatim transcripts and engaged in collaborative coding to minimise interpretive bias, subtle linguistic and cultural nuances may not have been fully captured. Despite the involvement of a local isiZulu-speaking translator, the positionality of the primary researcher as a white English-speaking woman may have shaped the dynamic of the interviews.

## Conclusion

This study provides insight into the complexity of treating a disease with an unknown cause when community perspectives are at odds with biomedical frameworks. We identified that barriers to treatment-seeking for MJD are multifaceted, arising from the interplay of treatment practices, aetiological beliefs and systemic constraints, including access to care and the role of family caregivers. These findings highlight that systemic and individual barriers are closely linked, and that improving disease management and patient outcomes requires addressing the concerns and experiences of both healthcare providers and patients. Targeted interventions that consider these intersecting barriers, particularly culturally appropriate education, physiotherapy support, and strengthened care provision by family members such as *uMakotis*, could have a meaningful impact on mobility, pain management, and overall quality of life for patients with MJD.
